# Impact of *ao-dake-humi*, Japanese traditional bamboo foot stimulator, on lower urinary tract symptoms, constipation and hypersensitivity to cold: a single-arm prospective pilot study

**DOI:** 10.1186/s12906-016-1494-1

**Published:** 2016-12-09

**Authors:** Tomonori Minagawa, Tetsuichi Saitou, Toshiro Suzuki, Takahisa Domen, Hitoshi Yokoyama, Masakuni Ishikawa, Shiro Hirakata, Takashi Nagai, Masaki Nakazawa, Teruyuki Ogawa, Osamu Ishizuka

**Affiliations:** Department of Urology, Shinshu University School of Medicine, 3-1-1 Asahi, 390-8621 Matsumoto, Nagano Japan

**Keywords:** *ao-dake-humi*, Lower urinary tract symptoms, Constipation, Hypersensitivity to cold, Urinary bladder, Colon, Skin, Neuromodulation, Reflexology

## Abstract

**Background:**

*Ao-dake-humi* is a traditional Japanese bamboo foot stimulator consisting of a half-pipe-shaped step made of bamboo used to stimulate the foot by stepping on it, and is commonly used to promote general health among the elderly in Japan. However, its efficacy has not been reported in the scientific literature. This study was performed to investigate the role of *ao-dake-humi* focusing on lower urinary tract symptoms (LUTS), constipation, and hypersensitivity to cold (HC).

**Methods:**

Participants with LUTS, constipation, or HC were enrolled in this study. *Ao-dake-humi* was used twice a day for 28 days. Before and 28 days after starting *ao-dake-humi* use, international prostate symptom score (IPSS), quality-of-life (QoL) score, and overactive bladder symptom score (OABSS) were measured to evaluate the efficacy of *ao-dake-humi* on LUTS. To evaluate the objective efficacy of *ao-dake-humi* on LUTS, a frequency-volume chart (FVC) was plotted in LUTS patients for 3 days. A visual analogue scale (VAS) was used to evaluate the efficacy of *ao-dake-humi* on constipation (VAS-constipation) and HC (VAS-HC) in the participants with constipation or HC.

**Results:**

A total of 24 participants were enrolled in this study. Twenty-one participants had LUTS, 11 had constipation, and 17 participants had HC. IPSS, especially storage-subscore, QoL score and OABSS, decreased significantly after use of *ao-dake-humi*. The use of *ao-dake-humi* increased maximal bladder capacity, resulting in a significant decrease in urinary frequency as determined from the FVC. In accordance with the results of VAS-constipation and VAS-HC, both constipation and HC were significantly relieved after *ao-dake-humi* use.

**Conclusion:**

The results of this prospective pilot study indicated that *ao-dake-humi* is safe and has therapeutic efficacy in cases of LUTS, constipation and HC. The possibility of using *ao-dake-humi* as physical neuromodulation therapy was shown in the management of LUTS, constipation and HC.

**Trial registration:**

UMIN000019333 (UMIN-CTR, Registered October-15-2015) retrospectively registered.

## Background

**Fig. 1:**
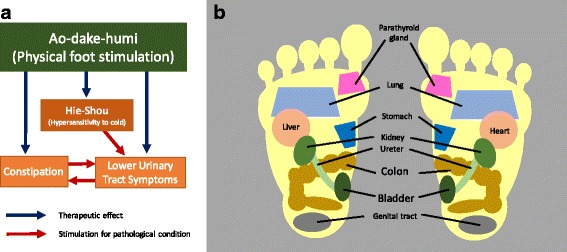
**a** Relationship among urinary bladder, bowel, and skin sensation. Interactions of pelvic organs and skin sensation on pathological condition are briefly presented. *Ao-dake-humi* can be a therapeutic stimulator for management via simultaneous and indirect effect on lower urinary tract symptoms, constipation, and hypersensitivity to cold. This figure is modified schema shown in Ref #2. **b** A brief foot chart in reflexology. Only main and large areas are shown in this figure, and small parts are excluded for ease of understanding. Each area of the foot matches specific body parts, and is connected to promoting health of each part of the body by massage. The colon and urinary bladder are located in the arch area, which is the “hot spot” stimulated by *ao-dake-humi*

Lower urinary tract symptoms (LUTS) and constipation are common problems, especially among the elderly [1]. On the other hand, skin sensation, especially to cold temperature, can be related to urinary bladder function. This symptomatic phenomenon is called “Hie-Shou” in Kampo-medicine (Japanese traditional medicine). Hie-Shou can be translated as hypersensitivity to cold (HC) [2]. HC induces several secondary symptoms, such as urinary urgency [2, 3]. Previously, we reported the importance of simultaneous management of LUTS, constipation and HC considering interaction among pelvic organs and skin [2] (Fig. 1a). On the other hand, foot massage and reflexology, are candidates for management of LUTS, constipation and HC [4–7]. The theory behind reflexology is that specific areas of foot match and are connected to specific organs of the body as shown in Fig. 1b. In Japan, a traditional bamboo foot stimulator called *ao-dake-humi* is commonly used to promote general health, and can be applied for management of LUTS, constipation, and HC as shown in Fig. 1a, b. However, the exact role of *ao-dake-humi* has not been proven. Therefore, we attempted to evaluate the safety and therapeutic efficacy of *ao-dake-humi* on LUTS, constipation and HC in this prospective pilot study.

## Methods

This study was conducted between July 2013 and October 2015. The trial registration number is UMIN000019333 (UMIN-CTR, registration date is 15th Oct. 2015).

Eligible participants were >20 y.o. with LUTS, constipation and/or HC. LUTS patients with IPSS >13 or dissatisfied with current LUTS treatments were enrolled. Constipation and HC were evaluated according to self-description. Exclusion criteria were as follows: patients who could not make the decision themselves, those with a custom of receiving foot massage, those incapable of walking by themselves, and those with a history of recurrent urinary tract infection or bladder stones. All of the LUTS patients had already received some medical care in a hospital or a private clinic. In the process of the previous management of LUTS before enrolling this trial, urinary tract infection and cancer including prostate cancer had already excluded. Subjects were also excluded if they received surgery, diagnosed malignant disease, and used other medical interventions, such as drugs, within the previous 3 months. Participants could continue to take medication for LUTS and constipation to maintain the same condition before and after the study. However, the participants were not permitted to start or stop any intervention for LUTS, constipation or HC during the study.

**Fig. 2:**
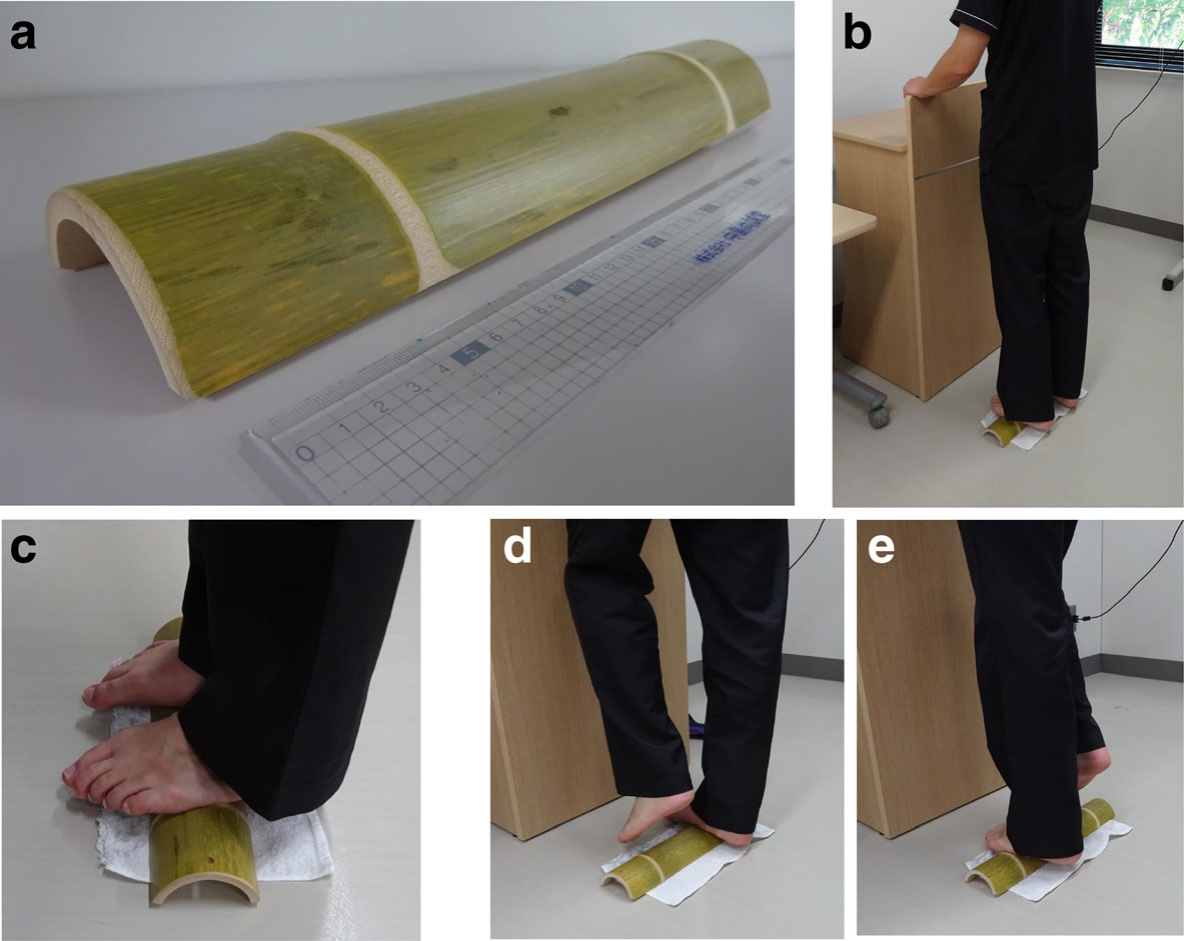
**a** Photo of *ao-dake-humi*. **b**, **c**, **d** Use of *ao-dake-humi*. **b**, **c**, **d**, **e** Holding something such as a wall, desk, or pillar, participants place both feet on *ao-dake-humi*, and then make repeated steps for 2 min in a set

*Ao-dake-humi* used in this study was made of bamboo, 40 cm in length, 8.5 cm in width, 4.5 cm in height, and almost 290 g in weight. The *ao-dake-humi* was brought to each participant’s home, and used twice a day, in the morning (after waking up) and evening (after taking a bath/shower or before going to bed) for 28 days. Holding something such as the wall, desk, or pillar, participants placed the arches of both feet on the *ao-dake-humi*, and then made repeated steps for 2 min in a set as shown in Fig. 2. Figure 2 includes the photographs with one of the author (T.M.). In accordance with the usual use of *ao-dake-humi*, steps were made 30–60 times/min, and participants could control the rate by themselves. If participants felt pain, they could reduce use to once a day. The rate of *ao-dake-humi* use was noted using a diary to evaluate the exact intervention and harmfulness of *ao-dake-humi*. All of the participants recorded *ao-dake-humi* use as “done” or “none” in the diary. Total practical rate (= number of sets performed/(2 sets  ×  28 days)) was calculated in all of the participants. Moreover, adverse events were also evaluated in all of the participants during this research.

Before and 28 days after *ao-dake-humi*, international prostate symptom score (IPSS), quality-of-life (QoL) score, and overactive bladder symptom score (OABSS) were measured for evaluation of efficacy of *ao-dake-humi* on LUTS. IPSS storage, voiding, and postvoiding subscores were also assessed [8, 9]. Visual analogue scale (VAS) was used to evaluate the efficacy of *ao-dake-humi* on constipation (VAS-constipation) and HC (VAS-HC) in the enrolled participants with constipation or H*C.* VAS for constipation and HC were originally made, and were used to evaluate constipation and HC like pain scale. The line is 10 cm in length on the paper. The left edge of line is zero, and the right edge is maximal as 10 points. Zero means that patients have not any symptoms. And, 10 points means that patients have severe symptoms which patients can imagine maximally. Patients can place a vertical mark on the line to describe the severity of their symptoms intuitively. If the patients have severe symptoms, patients can put on the right part of the line. The length from zero to the point on which patients described the symptoms is treated as severity of symptoms, and the decrease or increase of length are considered as improvement or worsening of symptoms. IPSS, QoL score, OABSS, VAS-constipation, and VAS-HC was presented in Table 1 (written in English).

**Table 1:**
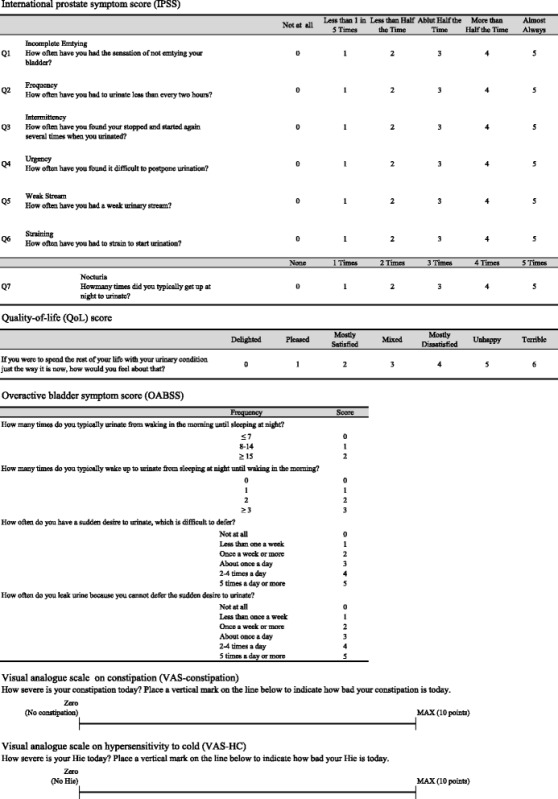
Medical interview sheet to evalutate lower urinary symptoms (LUTS) and visual analogue scale (VAS) to evaluate constipation and hypersensitivity to cold (HC). International prostate symptom score (IPSS), quality of life (QoL) score, and overactive bladder symptom score (OABSS) were used for LUTS. VAS were originally made to evaluate constipation and HC. Zero means that patients have not any symptoms. And, 10 points means that patients have severe symptoms which patients can imagine maximally

To evaluate the objective efficacy of *ao-dake-humi* on LUTS, a frequency-volume chart (FVC) was used. The FVC on 3 continuous days was recorded in all of the enrolled LUTS participants before and 28 days after starting *ao-dake-humi* use. The average total voided volume, daytime voided volume, nighttime voided volume, total urinary frequency in a day, daytime urinary frequency, and nighttime urinary frequency were evaluated for each day. The average mean voided volume, maximal voided volume, and minimal voided volume were also evaluated. Nighttime micturition was defined as micturition between 10:00 pm and 6:00 am.

All data are presented as the means ± SEM. The data were analysed using Excel-based statistical program file, Excel TouKei (Social Survey Research Information Co., Ltd., Tokyo, Japan), and *p* < 0.05 was considered to indicate significance. The changes before and after *ao-dake-humi* were analysed using the paired *t* test.

## Results and discussion

A total of 24 participants were enrolled in this study, and their characteristics are shown in Table 2. The results of subjective evaluation are shown in Table 3. The baseline of total IPSS, QoL score, and OABSS among LUTS patients is 14.7 ± 8.2, 4.3 ± 1.3, and 5.4 ± 3.6, respectively. Total IPSS, OABSS, and QoL score were improved after *ao-dake-humi* use in the participants with LUTS. In analysis of IPSS subscores, storage and postvoiding symptom subscores were significantly improved after *ao-dake-humi* use. As the first step to show the efficacy of *ao-dake-humi* on constipation and HC, evaluation was performed in this study using only VAS-constipation and VAS-HC. In accordance with the results, of the 11 participants with constipation, subjective VAS-constipation score was improved in −31.8% after *ao-dake-humi* use with statistical significance. Of the 17 participants with HC, subjective score was significantly improved in −21.9% of VAS-HC after *ao-dake-humi* use. The results of objective evaluation are shown in Table 4. Total urine volume did not change before and 28 days after starting *ao-dake-humi* use. Total and daytime urinary frequencies were decreased after *ao-dake-humi* use. These results may have been due to an increase in voided volume. Only the maximal voided volume increased significantly after *ao-dake-humi* use; mean and minimal voided volumes were also not significantly increased.

**Table 2:** Participant characteristics

Total		
Sex		
	Male	
	Female	
Age	(y.o.)	65.2 ± 16.3
Body mass index (kg/m 2)		21.8 ± 2.2
Distribution of LUTS, constipation, and HC		
	LUTS	21
	Constipation	11
	HC	17
	Only LUTS	5
	Only Constipation	0
	Only HC	1
	LUTS and constipation	2
	LUTS and HC	7
	Constipation and HC	2
	LUTS, constipation, and HC	7
Past history		
	Benign prostatic hyperplasia	8
	Hypertention	3
	Diabetes mellitus	1
Medication		
	Alpha 1-adrenocepter antagonist	8
	Anticholinergic drug	1
	Beta 3-adrenoceptor agonist	3
	Cathartic drug	5

Characteristics of the enrolled patients in this study

*LUTS* lower urinary tract symptoms, *HC* hypersensitivity to cold

**Table 3:** Differences in lower urinary tract symptoms, constipation and hypersensitivity to cold (HC) before and after *ao-dake-humi* use

LUTS			LUTS (*N* = 21)	*p* value
	IPSS	Total	−3.8	0.0002**
		Voiding subscore	−1.0	0.0592
		Storage subscore	−2.1	0.0000**
		Postvoiding subscore	−0.7	0.0060**
	QOL score		−1.2	0.0000**
	OABSS		−1.1	0.0070**
Constipation			Constipation (*N* = 11)	
	VAS-constipation		−31.8	0.0073**
HC			HC (*N* = 17)	
	VAS-HC		−21.9	0.0158*

*LUTS* lower urinary tract symptoms, *IPSS* international prostate symptom score, *QOL* quality of life, *OABSS* overactive bladder symptom score, *HC* hypersensitivity to cold, *VAS* Visual analogue scale

Subjective effect of ao-dake-humi on lower urinary tract and constipation and hypersensitivity to cold. Different before and after ao-dakehumi was shown. *: *p* < 0.05, ** *p* < 0.01

**Table 4:** Objective values before and after *ao-dake-humi* based on frequency-volume-chart (FVC)

	Before	After	*p* value
LUTS (*N* = 20)			
Total urine volume (ml/day)	1684.7 ± 788.5	1669.1 ± 691.2	0.4543
Mean voided volume (ml)	169.7 ± 52.8	187.0 ± 67.0	0.0538
Maximal voided volume (ml)	331.5 ± 140.3	373.0 ± 139.0	0.0424*
Minimal voided volume (ml)	58.3 ± 31.4	72.8 ± 50.2	0.0948
Total urinary frequency (/day)	10.9 ± 3.5	9.8 ± 2.7	0.0065**
Day-time urinary frequency (/6–22 o’clock)	8.9 ± 2.7	7.9 ± 1.7	0.0162*
Night-time urinary frequency (/22–6 o’clock)	2.0 ± 1.7	1.8 ± 1.6	0.1898

Objective results of ao-dake-humi on LUTS. One of 21 patients did not make ao-dake-humi-diary

LUTS: lower urinary tract symptoms. *: *p* < 0.05, ** *p* < 0.01

Previous reports indicated that a decrease in environmental temperature induces numerous physiological responses, including increased urinary frequency in both humans and animals [10, 11]. Previously, we reported the relationship between HC and LUTS, and the efficacy of simultaneous approaches using Kampo-medicine [2]. In this study, 60% of the enrolled participants had both LUTS and HC, and the efficacy of *ao-dake-humi* on HC was revealed subjectively. Moreover, constipation was also improved after *ao-dake-humi* use. Five of 11 participants with constipation had received drug treatment before starting this research. These results indicated that *ao-dake-humi* is a candidate therapeutic option to manage LUTS, constipation and HC. However, HC and constipation were evaluated on the basis of self-description using VAS. A further RCT with larger number of participants is needed.

*Ao-dake-humi* is a Japanese traditional home health appliance based on the theory of reflexology, and can be used at home to promote general health in daily use. *Ao-dake-humi* consists of a half-pipe-shaped foot-step made of bamboo cut into pieces 30–40 cm in length, and is widely used in Japan. The mechanisms of *ao-dake-humi* promoting general health are not yet known. However, neuromodulation and acupuncture may have similar mechanisms of action on LUTS, constipation and HC. Therefore, the effect of *ao-dake-humi* is thought not to be due to exercise but to stimulation of afferent nerves in the foot to modulate visceral organs, such as the urinary bladder and colon. Indeed, some neuromodulation equipment has been reported for treatment of LUTS, constipation and faecal incontinence [12]. Electrical stimulation [13–18], magnetic stimulation [19–22] and interferential therapy [23, 24] are mainly used for modulation via the sacral nerve or tibial nerve. Although the precise mechanisms of neuromodulation are not yet known, activation of afferent nerves may lead to activation of efferent nerves in the pelvic organs, including the urinary bladder and colon [13, 14]. Physical stimulation, such as acupuncture, reflexology or foot massage, can be used as a source of neuromodulation via activation of sensory afferent pathways. In addition, the theory of reflexology, including acupuncture, can also be adapted for *ao-dake-humi,* and our hypothesis about *ao-dake-humi* is described on Fig. 1a also. In reflexology, areas of the foot are believed to correspond to the organs or structures of the body [25]. Acupuncture was reported to have be effective for LUTS and constipation [26–30]. Moreover, in accordance with the foot chart of oriental foot massage, the urinary bladder and colon are located in the “hot spot” of *ao-dake-humi* stimulation (Fig. 1b). Of course, the exercise effect of *ao-dake-humi* cannot be completely excluded in this study. Therefore, an RCT should be done between step-work-exercise versus *ao-dake-humi*.

*Ao-dake-humi* has many advantages, such as its inexpensiveness, portability, and less risk of harm, compared to other interventions, including electrical neuromodulation. *Ao-dake-humi* costs about 5 dollars, and requires little maintenance. Moreover, as *ao-dake-humi* can be shared, it can be used in public spaces, such as hospitals and nursing homes. *Ao-dake-humi* is lightweight (almost 300 g), and is therefore easily portable, and ideal for daily use at home. Even in comparison with other similar treatments, including acupuncture and massage, *ao-dake-humi* has advantages. For example, the amount of stimulation can be controlled procedure-dependently in normal foot massage. In contrast, the amount of stimulation of *ao-dake-humi* is controlled according to bodyweight and of each patient. The average body mass index of the participants was 21.8 kg/m^2^, and obese participants were not included in this study. Although adequate methods should be proposed for participants with obesity in future studies, *ao-dake-humi* can standardise the amount of stimulation for patients. Adverse events associated with neuromodulation, such as infection, bleeding, pain, etc., have been reported [31, 32]. In this study, only three participants complained of slight pain due to *ao-dake-humi* use, and 98.5% of participants achieved use of *ao-dake-humi* according to a review of their diaries. All of the participants wished to continue *ao-dake-humi* use after this study. Even the participants that complained of slight pain at commencement of this study eventually felt comfortable with *ao-dake-humi* stimulation. There were no severe adverse events, such as diarrhea or urinary retention, among the participants. Of course, on theoretical grounds, it is impossible for the participants to have any severe advised events associated with use of *ao-dake-humi*. Compared with other neuromodulators, this is the unquestionable advantage of *ao-dake-humi* in management of LUTS and constipation.

However, this study had some limitations. In this pilot study, 2 sets/day of 2 min of *ao-dake-humi* were done for 28 days. There have been no reports regarding adequate use of *ao-dake-humi*. Time, duration, and frequency should be evaluated in future studies. The most important limitation of this study is bias because of its single-arm nature. Subjective results, such as IPSS and OABSS, should be interpreted carefully. However, objective results from FVC indicated that *ao-dake-humi* has efficacy for LUTS. Of course, objective evaluation should be performed even for constipation and HC, and more objective evaluation for LUTS, including uroflowmetry, residual urine volume, and cystometry, should also be performed in future studies. Lack of objective evaluation of constipation and HC is also a limitation of this study. However, object evaluation for constipation without any intervention is difficult, and object measurement of HC has not been established yet. Further investigation is needed to evaluate the exact role of *ao-dake-humi* on constipation and HC. However, even with these limitations, *ao-dake-humi* showed statistically relevant subjective and objective results in this study, and there were no adverse events. Thus, *ao-dake-humi* is a candidate of therapeutic option for LUTS, constipation and HC.

## Conclusions

The results of this pilot study indicated that *ao-dake-humi* improved subjective symptoms of LUTS, constipation and HC. *Ao-dake-humi* also increased maximal voided volume leading to a reduction in urinary frequency without any severe adverse events.

## Abbreviations

FVC: Frequency-volume-chart
HC: Hypersensitivity to cold
IPSS: International prostate symptom score
LUTS: Lower urinary tract symptoms
OABSS: Overactive bladder symptom score
QoL: Quality of life
